# Influence of Repetitive Square Voltage Duty Cycle on the Electrical Tree Characteristics of Epoxy Resin

**DOI:** 10.3390/polym12102215

**Published:** 2020-09-27

**Authors:** Peng Wang, Suxin Hui, Shakeel Akram, Kai Zhou, Muhammad Tariq Nazir, Yiwen Chen, Han Dong, Muhammad Sufyan Javed, Inzamam Ul Haq

**Affiliations:** 1 College of Electrical Engineering, Sichuan University, Chengdu 610065, China; pwang@scu.edu.cn (P.W.); hsx1759499817@163.com (S.H.); zhoukai_scu@163.com (K.Z.); ceven96@163.com (Y.C.); major2011@163.com (H.D.); 2 School of Mechanical and Manufacturing Engineering, University of New South Wales, Sydney, NSW 2052, Australia; muhammadtariq.nazir@unsw.edu.au; 3 Department of Physics, Jinan University, Guangdong 510632, China; safisabri@gmail.com; 4 State Key Laboratory of Power Transmission Equipment & System Security and New Technology, Chongqing University, Chongqing 400044, China; inzimam.324@gmail.com

**Keywords:** epoxy resin, electrical tree, duty cycle, power electronic devices, square wave voltage, electric machines insulation

## Abstract

The application of wide band-gap power electronic devices brings more challenges to insulating packaging technology. Knowing the influence of applied voltage parameters on insulation performance is helpful to evaluate the insulation condition of electric power equipment. In this paper, the effect of repetitive square wave voltage duty cycle on the growth characteristics of electrical trees in epoxy resin was studied. The experimental results show that the square wave voltage duty cycle has a significant influence on treeing features. The electrical tree proportion initiation has shown a decreasing trend, and the shape of the electrical tree changes from pine-like to branch-like by increasing the duty cycles. The length and damaged area of electrical tree increased with the increase in the duty cycle up to 10% and then decrease by increasing the duty cycle higher than 30%. It indicates that a low duty cycle will enhance the electron injection and accumulate space charges and thus accelerate electrical tree development. Under short duty cycles, the electric field due to the shielding effect near the needle tip suppresses the electrical tree growth, which results in treeing growth stagnation. The obtained results are helpful to keep these parameters in mind during the design of epoxy-based insulation such high-voltage rotating machines and power electronic device packaging.

## 1. Introduction

Electric power equipment is the expensive and core parts of electric power systems. The application of third-generation wide band-gap semiconductor devices, represented by silicon carbide (SIC) and gallium nitride (GAN), enables the existing power electronic system to work at the high switching frequency and voltage level, thereby requiring high insulation reliability for such power electronic devices [1,2,3]. Packaging technology determines the reliability of power electronic devices. The high-frequency and high-voltage stresses generated by wide band-gap devices bring more challenges to the traditional insulation technology and materials used in silicon-based semiconductor devices [4,5,6].

Epoxy resin has been used as packaging materials in power electronic devices because of its excellent physical and mechanical properties, chemical resistance and electrical insulation [7,8,9]. Pulse width modulation (PWM) technology is a standard transformation technology of power electronic devices that can produce sinusoidal current waveform on the load by changing the duty cycle. The duty cycle is the primary parameter of PWM technology, and thus the performance of insulation packaging materials under different duty cycles should be considered carefully. The electrical stresses on epoxy resin generated by the repetitive impulsive voltage are different from traditionally operated sine wave voltage and DC voltage conditions. Therefore, the performance of epoxy resin under square wave voltage by varying duty cycles is the primary consideration for its application as the insulation packaging of power electronic devices to improve the safety, stability, and reliability of the insulation system.

The electrical tree is an essential phenomenon of physical defects in epoxy resin, which can lead to the electrical breakdown of insulating materials under high electric field conditions. To avoid the micro defects entirely in the solid insulation packaging material is difficult because of the limitations in the industrial production process [10,11]. These physical defects may undergo mechanical and chemical changes under electrical, thermal, and mechanical stresses. Once the electric field is localized at a certain point, the partial discharge may be produced to generate further electrical trees [11,12]. The growth of the electrical tree eventually leads to the breakdown of insulating materials, which seriously threatens the security and stability of the power electronic system.

The initiation and growth characteristics of the electrical tree in epoxy resin have been investigated in recent years. In Reference [13], the experimental results indicate that, when the DC and pulse voltage are of the same polarity, the rate of tree growth increases with the amplitude of DC voltage rising but would slow down after a certain period. When the polarity of DC and pulse voltage is opposite, the growth rate decreases with the DC voltage amplitude increasing. A polarity dependence of treeing at DC voltage was studied in [14], showing that the morphology of the initial trees, either bush-like or branch-like, does not have a pronounced effect on the structure of the subsequent DC tree. The impact of combined DC-harmonic voltage ranging from −20 to +20 kV was investigated in epoxy resin [15]. It was reported that the tree structure tends to be branch-like with positive DC voltage and has a relatively high probability of being bush-branch like with negative DC voltage. None of the above research can fully reflect the insulation performance of epoxy resin under high-frequency square wave voltage conditions by the varying duty cycle. Therefore, it is necessary to investigate the growth characteristics of the electrical tree under repetitive PWM voltage. 

From the previous research, we have found that the square wave voltage parameters have a significant influence on the failure process of turn to turn insulation for inverter-fed motors [16,17,18]. The partial discharge tested performed on the turn to turn insulation at square wave voltages with different duty cycles, showing that the smaller or higher duty cycle would cause the asymmetry of the partial discharge patterns, thereby affect the insulation life. This conclusion is only applicable to the evaluation of corona resistance turn-to-turn insulation of inverter-fed motors. 

This paper aims to report our study on the influence and mechanism of repetitive square wave voltage duty cycles (100%× positive voltage duration/square wave voltage cycle time) on the electrical tree growth characteristics of epoxy resin while keeping the peak-to-peak magnitude, frequency, and rise time of square wave voltage at 12 kV, 1 kHz, and 70 ns, respectively. The mechanism of the electrical tree under square wave voltage is proposed by analyzing the treeing initiation proportion, shape, length and damaged area under different duty cycles. The results obtained in this study are expected to provide a reference for the design of insulation package, the evaluation of insulation reliability, and the improvement of insulation performance in power electronic systems.

## 2. Experimental Setup

### 2.1. Specimen Preparation and Treeing Test Platform

**Figure 1:**
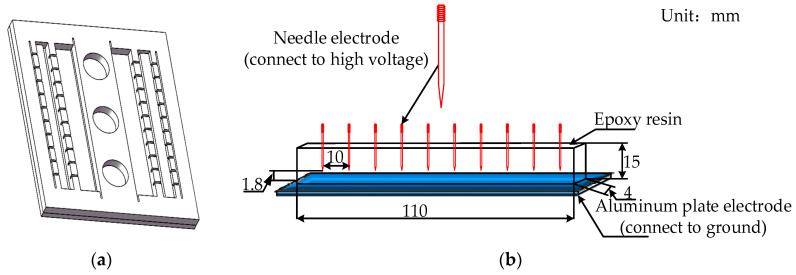
(**a**) Epoxy resin casting mold; (**b**) needle-plate electrode sample.

E-51 epoxy resin and methyltetrahydrophthalic anhydride curing agent are mixed and solidified in a 2:1 mass ratio. The epoxy resin and curing agents are preheated before mixing. The two agents are mixed and stirred clockwise for 30 min at 60 °C. The mould with the size of 120 × 120 × 10 mm^3^ was used for sample preparation (see Figure 1a). The needle electrode and aluminum plate were embedded in the epoxy resin in advance to prevent bubbles in the sample. The mixed liquid after vacuum defoaming is poured into the mould using the needle electrode placed in advance and placed into the vacuum drying oven. The curing time is 24 h, keeping the temperature at 60 °C. In Figure 1b, the needle electrode size 33 mm length and 180 μm diameter with model specification of 06cr19ni10 (SUS304) stainless-steel wire is used. The curvature e radius of the needle tip is 3 ± 0.2 μm, the surface roughness less than 0.63 μm, the included angle of the needle tip 30°, and the distance between the needle tip and the grounding aluminum plate is 1.8 ± 0.1 mm.

**Figure 2:**
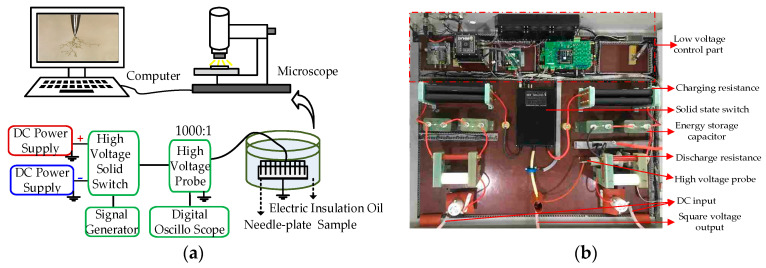
Testing platform for the growth of the electrical tree. (**a**) Schematic diagram and a (**b**) physical picture of square wave power supply.

The test platform for electrical tree growth is shown in Figure 2. The square wave voltage generator consists of a positive and negative DC power supply, signal generator, high-voltage solid-state switch and the low voltage control part. The parameters of the square wave voltage can be seen in Table 1, and all parameters can be adjusted. The peak-to-peak magnitude and polarity of the output voltage could be adjusted by the positive and negative DC power supply. The duty cycle and frequency could be adjusted by changing the parameter of the signal generator, which was used to control the switching on and off of the high-voltage solid-state switch module to produce high-voltage square wave voltages. If we change the charging resistance and energy storage capacitor, the rise time of the output voltage can be changed from ns to ms level.

In this experiment, the needle-plate sample was immersed in electric insulation oil to avoid corona discharge at the high-voltage output. The needle electrodes were connected to the high-voltage output terminal, and the aluminum plate electrode was grounded. The output voltage signal was monitored by using a high-voltage probe (Tektronix P6015A, 50 MHz frequency bandwidth, Beaverton, OR, USA) and transmitted to a digital oscilloscope for observation. The parameters of the electrical tree were recorded and observed by a high-precision metallographic microscope (Leica DVM6, Wetzlar, Germany). 

### 2.2. Experimental Procedure

Fifteen needle-plate electrode samples were selected to measure the initiation proportion and a total of 10 pin electrode samples were tested the propagation under the same duty cycle to reduce the possible influence of sample randomness on the experimental results. As shown in Figure 2, treeing tests were performed at square wave voltages with an adjustable duty cycle from 5% to 90% while keeping the peak-to-peak voltage, frequency, and rise time at 12 kV, 1 kHz, and 70 ns, respectively. Then, the treeing parameters (such as length and shape) were recorded by the microscope.

## 3. Results

The length of the electrical tree is defined in this paper as the longest distance in parallel direction from the needle tip to the end of electrical tree and the width of the electrical tree as the horizontal longest distance from one end of the electrical tree to the other end.

### 3.1. Effect of Duty Cycle on the Treeing Initiation Proportion

**Figure 3:**
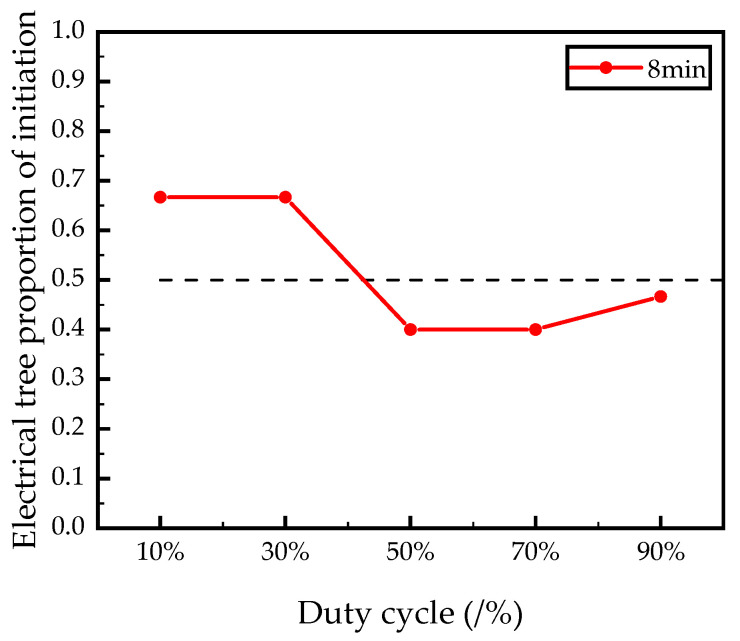
Electrical tree initiation proportion versus testing time under square wave voltage with different duty cycles (12 kV/1 kHz).

The electrical tree initiation is defined when the tree length is longer than 20 μm [19]. Table 2 is showing the probability of treeing initiation at two levels of time under different duty cycles. The number of electrical tree initiations changes by varying duty cycles, as shown in Table 1. At the short duty cycle, the chances of tree initiation are higher than that under high duty cycles with the treeing time increasing. From Figure 3, we can see a falling slope of the electrical tree initiation proportion. Taking 0.5 as the reference line, it can be concluded that when the initiation proportion of electrical tree is greater than 0.5, there would be a higher possibility of initiating electrical trees in a short time. When the electrical tree initiation proportion is less than 0.5, there would be less possibility of initiating electrical trees in a short time. Therefore, square voltage with short duty cycle (long duration of negative voltage) is more likely to cause the initiation of the electrical tree.

### 3.2. Electrical Tree Morphology and Growth Characteristics

**Figure 4:**
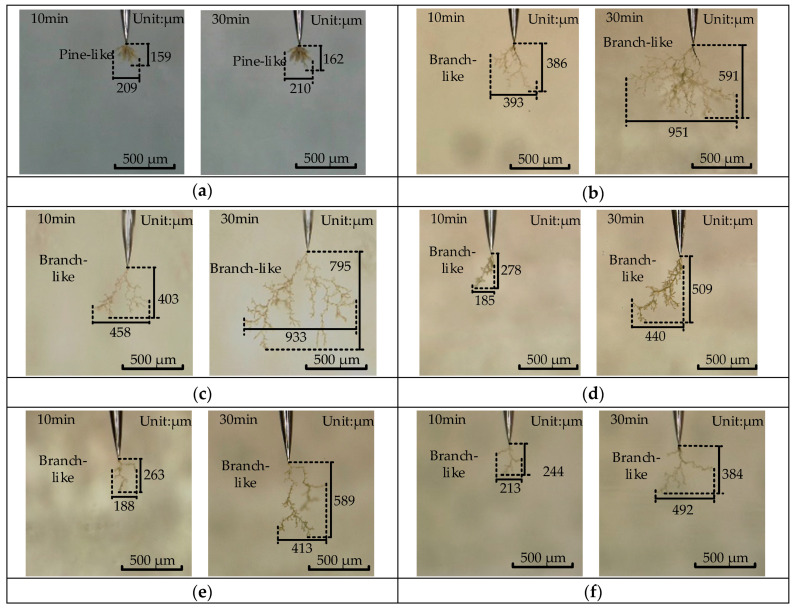
Typical morphology of electrical tree under square wave voltage with different duty cycles. (**a**) 5% duty cycle, (**b**) 10% duty cycle, (**c**) 30% duty cycle, (**d**) 50% duty cycle, (**e**) 70% duty cycle, (**f**) 90% duty cycle.

Figure 4 shows the typical shape of the electrical tree under square wave voltage with different duty cycles. The number and length of branches increased with treeing time under the same duty cycle. The typical shapes of the electrical tree change from pine-like to branch-like.

At 5% duty cycle, the electrical tree branches are dense in the form of pine-like structure and basically did not change in shape within 10–30 min. At 10% duty cycle, many branches of the electrical tree and the end of the electrical tree tend to be hemispherical with the increase in time. Under 30%, 50%, 70%, and 90% duty cycles, the channel of the electrical tree decreased, and the electrical tree became sparse with the increase in duty cycle.

**Figure 5:**
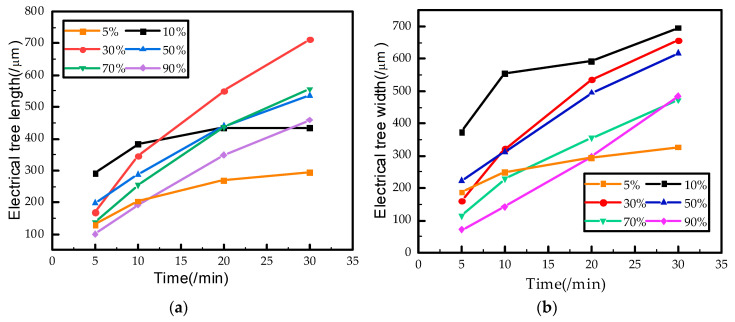
Electrical tree growth versus testing time under square wave voltage with different duty cycles. (**a**) Electrical tree length versus testing time and (**b**) Electrical tree width versus testing time.

Figure 5 shows the relationship between the length and width of the electrical tree and the square wave voltage duty cycle. It can be seen that the electrical tree length tends to decrease by increasing the square wave voltage duty cycles higher than 10%. Both the growth rate of length and width are linear within 30 min. And the growth rate of the electrical tree at 30% duty cycle is the fastest, and the average length of the electrical tree reached 712 μm and the maximum length was 983 μm at 30 min.

However, the growth rate of the electrical tree for 5% and 10% duty cycles began to slow down after 10 min. The length and width of the electrical branch are significantly reduced when the duty cycle was reduced to 5%. Comparison of Figure 4a,b shows that electrical branches are dense under 10% and 5% duty cycles, the shape of the electrical branches did not change much within 10–30 min, and a growth stagnation period occurred. The growth of electrical tree could be inhibited to a certain extent when the duty cycle is smaller than a specific value. However, the threshold value of duty cycle (duration of positive and negative voltage) for the growth stagnation period of electrical tree remains to be further studied.

### 3.3. Electrical Tree Damaged Proportion

**Figure 6:**
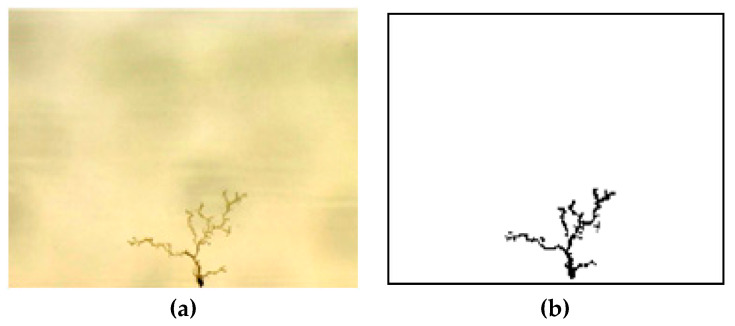
Example of electrical tree image binarization processing. (**a**) Electrical tree original image, (**b**) Electrical tree after image binarization processing.

The area of the electrical tree reflects the degree of damage to the epoxy resin caused by electrical tree branches. A fixed-size area (188 pixels × 151 pixels) is selected, where the damage proportion is defined as the proportion of the area of electrical tree to the area of the selection. As shown in Figure 6, a fixed size is selected for the electrical tree image under each duty cycle, and then the image is binarized to calculate the proportion of electrical tree damage [20,21].

**Figure 7:**
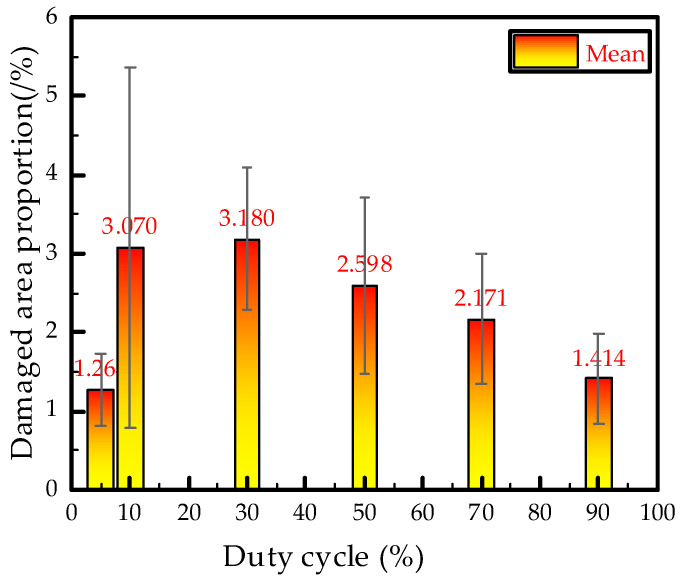
Damaged area proportion of electrical tree versus duty cycle under square wave voltage at 30 min.

Figure 7 is the mean deviation diagram of the damaged area proportion of electrical tree. The treeing damaged proportion gradually decreases with the increase of duty cycle when the square voltage duty cycles are higher than 10%, and the largest value of the damaged area lies in the 30% duty cycle group. Based on Section 3.2 and Section 3.3, a conclusion can be drawn that a small duty cycle (long duration of negative voltage) has a greater destructive effect on epoxy resin insulation materials.

However, the damaged area of the electrical tree under 5% duty cycle is smaller. Figure 4a shows that the electrical tree has a hemispherical pine-like structure at 5% duty cycle and so many electric branches have a certain shielding effect on the electric field intensity of the needle tip, hindering the growth of electrical tree. Therefore, the tree grows slowly and the area of damage to the material is small.

## 4. Discussion

**Figure 8:**
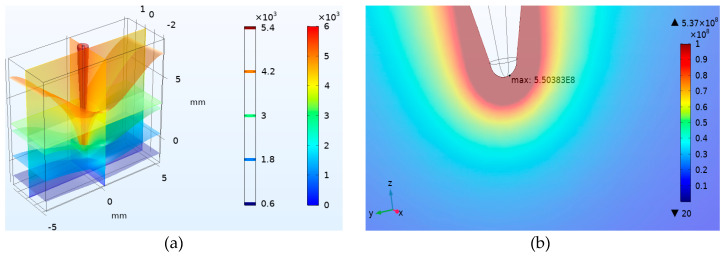
Electric field intensity distribution diagram of the needle-plate electrode. (**a**) Equipotential surface distribution and (**b**) maximum field intensity position.

To estimate the electric field intensity of the needle-tip electrode sample is crucial for electrical tree initiation, a simulation of the tip electric field was tested, as shown in Figure 8. The model parameters are consistent with the sample parameters used in the experiment, and the voltage of the needle electrode is +6 kV. It can be seen from Figure 8a that the electric field intensity near the needle electrode is obviously large, the maximum position is at the needle tip (see Figure 8b), and the maximum value is about 5.50 × 10^8^ V/m.

The electric field intensity at the tip of the needle used in the experiment can also be obtained from Equation (1) [22]:

(1) $$E=\frac{2V}{r\;\ln\left(4d/r\right)},$$

where *E* is the electric field near the needle tip, V the voltage amplitude at the needle tip of the sample, r the curvature of the tip, and d the distance between the needle tip and the plate electrode. In this study, *V*, *r* and *d* are ± 6 kV, 3 ± 0.2 μm and 1.8 ± 0.1 mm, respectively. The electric field intensity around the needle tip obtained by Equation (1) is approximately 5.14 × 10^8^ V/m.

Equation (1) is an approximate calculation equation. It can be seen that the calculated value 5.14 × 10^8^ V/m of the equation is quite close to the simulated electric field intensity value 5.50 × 10^8^ V/m, indicating that the extremely high electric field may reduce the potential barrier on the tip surface and result in the Fowler–Nordheim tunneling effect, which can induce electron emission from the needle tip [23,24]. The electron emission current density (*j*) under high electric field can be obtained by Equation (2) [25]:

(2) $$j=AT^{2}e^{\left[-\frac{\varphi -\sqrt{\frac{e^{3}E}{4{\pi \varepsilon }_{0}\varepsilon_{r}}}}{\left(\mathrm{kT}\right)}\right]},$$

where *A*, *T*, φ, *E*, ɛ_0_ɛ_r_ and *k* are the constant depending on metal parameters, metal temperature in Kelvin, metal work function, electric field of the needle tip, dielectric constant and Boltzmann’s constant. Obviously, the lnj is linearity to the $\sqrt{E}$ and thus the electron emission current density increases with increasing electric field intensity. Therefore, a higher electric field will give rise to a higher electron emission density.

**Figure 9:**
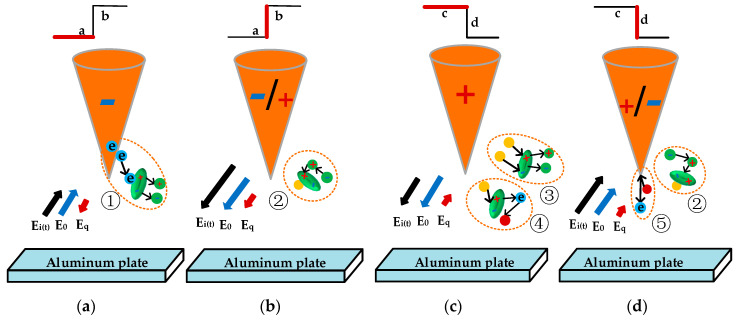
Change of space charge and electric field under square wave voltage. (**a**) Negative voltage, (**b**) rising flank, (**c**) positive voltage, (**d**) falling flank (elliptical green spheres: polymer molecules; green spheres: small molecule chains; yellow balls: photons; red balls: hot electrons).

**Figure 10:**
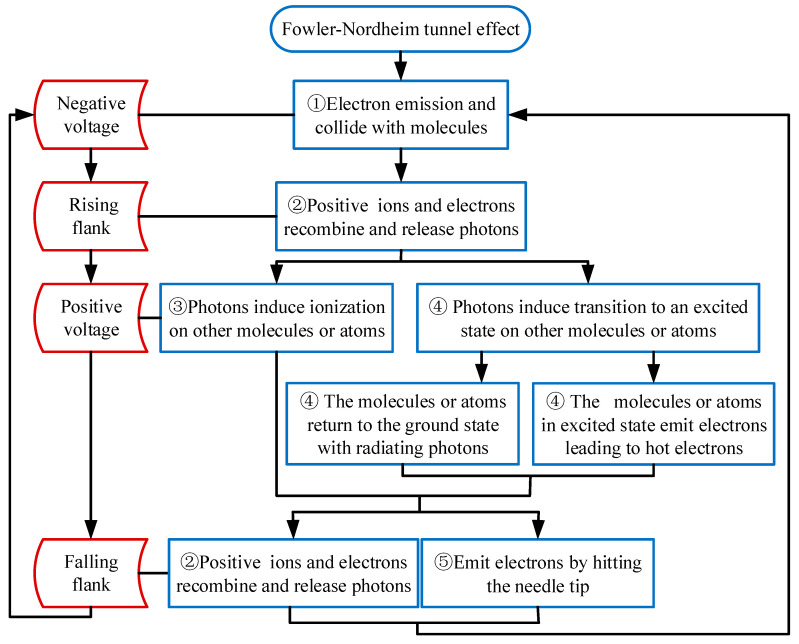
The flow chart of the process in Figure 9.

Figure 9 is showing the relation between space charges and the electric field near the tip of needle electrodes, where *a* is a negative voltage, *b* the rising flank, *c* the positive voltage and d the falling flank. *E*_0_ is the internal electric field due to external voltage and *E*_q_ the electric field produced by the space charge around the needle. *E*_i(t)_ is the total internal electric field, and e is an electron unit charge. Figure 9 is explaining the flow chart of these processes as mentioned in Figure 10.

In Figure 9a, the *E*_0_ higher than 10^8^ V/m could lead to a considerable Fowler–Nordheim tunnel effect (see Equation (1)) and induce the emission of electrons from the tip of the needle [24,25,26,27]. Ionization occurs when electrons with high energy collide with molecules in the polymer (process ①). In Figure 9b, the direction of the electric field of the space charge *E*_q_ in the polymer cannot change immediately just after the voltage polarity change at the rising flanks of square wave voltages. At this time, the applied electric field intensity *E*_0_ will have the same direction as the *E*_q_ and will result in the substantial increase of the total electric field *E*_i(t)_ [17]. As shown in Figure 9b,c, the electrons move toward the needle tip, causing the positive and negative ions to recombine and release the energy in the form of photons (process ②). These photons may induce more ionization (process ③) or transition to an excited state on other molecules or atoms near the tip. The unstable excited state would return to the ground state with radiating photons after around 10^−8^ s, or emit electrons by the Auger effect (process ④) and transfer energy to other electrons, leading to some of the electrons in the normal state changing to hot electrons [24,25].

In Figure 9c, the positive ions and electrons recombine and release photons and the needle tip emits a considerable number of electrons under negative voltage. On the one hand, it collides with polymer molecules and induces ionization (process ①) [28]. On the other hand, the photons generated by the recombination of positive ions and electrons could generate more emitted electrons (process ⑤) by hitting the needle tip (cathode) [29]. Both of these aspects damage the polymer molecular chain.

Energetic hot electrons may hit the polymer molecular chain, destroy and put the molecular chain into a free radical. The formation of free radicals in polymer would lead to a free-radical chain reaction and subsequently the generation of low-molecular products (e.g., hydroxyl, epoxy, etc.) [30,31]. The local area with the low-molecular products is called low-density regions [32]. Then, the average free path of electrons in the low-density region increases [13] and the small-molecule products in low-density regions are more prone to collision ionization. Finally, the ionization of polymer molecules is enhanced [33], which further aggravates the destruction of polymer molecular chains.

Accordingly, it can be summarized that the formation of hot electrons, ions recombination, and ionization by collision in low-density regions could damage the polymer molecular chain and form a conductive channel inside the polymer. Therefore, the electrical tree is generated near the tip of the needle. It can be seen from Table 2 that square wave voltages with smaller duty cycles (the duration of the negative voltage is greater than the positive voltage) lead to higher electrical tree initiation probability. A longer duration of negative voltage means more electrons will be injected into the polymer. As small-size electrons have a longer free path compared with ions, the kinetic energy of electrons is much greater than that of ions. Hence, electrons increase the probability of ionization by colliding with the polymer molecular chain, which would break the polymer molecular chain. Therefore, the electrical tree is easier to form under smaller duty cycles.

As the electrical tree branches of epoxy resin are conductive trees below the glass transition temperature [15], the electric field near the needle tip becomes extremely complicated as the electric branches grow. Equation (1) shows that, on the one hand, the existence of electric branches at the needle tip can shorten the distance between the high-voltage and ground electrode, resulting in an increased electric field intensity. On the other hand, these small branches would lead to a uniform electric field near the needle tip, weakening the electric field intensity. Eventually, the electrical tree grows substantially linearly, as shown in Figure 5.

As longer negative voltage duration (smaller duty cycle) induce more electrons near the needle tip. The electrons would collide with the epoxy resin molecular chain or its small molecule products and generate smaller molecular chains or free radicals, expanding the ionization area and forming a larger low-density region near the needle tip. Therefore, the length and the damaged area of electrical tree branches will increase under small duty cycles.

However, denser electric branches have a stronger weaken effect on the electric field [34], which is equivalent to increasing the needle tip curvature r in Equation (1). Thus, the growth of the electrical tree in the direction of the electric field was suppressed, and a growth stagnation period occurred to a certain extent under 5% and 10% duty cycles. And the range of damage to the epoxy resin is shortened under these duty cycles.

## 5. Conclusions

The statistical characteristics of electrical trees for epoxy resin using needle-plate electrodes were investigated experimentally at repetitive square wave voltage. The square wave voltage duty cycle has a considerable effect on the initiation and growth of electrical tree branches due to the total electrons emitted from the needle tip under positive and negative voltages. The following conclusions can be drawn from the study.

1. The number of electrical tree initiations varies by varying the duty cycles. The probability of tree initiation under small duty cycles is higher than that under high duty cycles with the treeing time increasing.
2. Repetitive square wave voltage duty cycle has a significant influence on the electrical tree morphology in epoxy resin. More electron emission and accumulation at square wave voltage with smaller duty cycles (longer negative voltage duration) could break molecular chains in epoxy with a higher probability. Moreover, the electrical trees develop from a pine-like into a dense branch-like structure and finally develop into a sparse branch-like structure at square wave voltages with increasing duty cycle.
3. When the duty cycle of the square wave voltage is higher than 30%, the electrical tree grows linearly and the electrical tree length, width and damage area decrease when increasing the duty cycles. However, the growth of the electrical tree may be suppressed when the duty cycle is less than 10%, due to the shielding effect of pine-like and dense branch-like structures, which generate hemispherical distribution near the needle-tip.

## Figures and Tables

**Table 1:** Parameters of square wave voltage.

Peak-to-Peak Magnitude	Polarity	Frequency	Duty Cycle	Rise Time
0–15 kV	Unipolar/Bipolar	0–5 kHz	0–100%	ns-ms level

**Table 2:** Number of tree initiation.

Duty Cycle	Number (3 min)	Number (8 min)	Total Number
10%	8	10	15
30%	9	10	15
50%	2	6	15
70%	3	6	15
90%	5	7	15
